# COPING STRATEGIES AND THEIR INFLUENCE ON DEPRESSIVE SYMPTOMS DURING THE SECOND COVID-19 PANDEMIC WAVE IN SWITZERLAND

**DOI:** 10.1093/geroni/igac059.2392

**Published:** 2022-12-20

**Authors:** Charikleia Lampraki, Daniela Jopp, Adar Hoffman, Angélique Roquet, Kim Uittenhove, Stefan Sieber

**Affiliations:** University of Lausanne, Lausanne, Vaud, Switzerland; University of Lausanne, Lausanne, Vaud, Switzerland; University of Lausanne, Lausanne, Vaud, Switzerland; University of Lausanne, Lausanne, Vaud, Switzerland; University of Lausanne, Lausanne, Vaud, Switzerland; LIVES Centre, University of Lausanne, Lausanne, Vaud, Switzerland

## Abstract

Coping strategies help individuals face stressful events and adapt to them. During the second wave of the COVID19 pandemic, individuals were confronted with increased governmental restrictions that aimed in impeding the propagation of the virus, but affected, at the same time, the life as we knew it, with negative consequences for mental health. This study aims at identifying the coping strategies that individuals used during this period, whether they changed over time and how they affected depressive symptoms in a life span sample in Switzerland. Our sample consisted of 736 individuals with age ranging between 18 and 81 years. The study was conducted in three waves with one-month intervals during the second pandemic-wave (i.e., October, November, December 2020). We used multilevel modelling to identify within-subject change and between-subject differences in depressive symptoms, with coping strategies and sociodemographic variables included as predictors. Older age, male gender, cohabiting with others, and being employed protected from feeling depressed. Results also indicated that seeking functional support, seeking emotional support, positive reappraisal and acceptance decreased, while self-distraction and depressive symptoms increased. When positive reappraisal decreased or/and when self-distraction increased, depressive symptoms also increased. This protective effect of positive reappraisal on depression differed in magnitude for younger and older individuals: Reduction in positive reappraisal was more strongly related to increases in depression for younger individuals. In sum, to adapt to the pandemic stress individuals changed the frequency of coping strategy use, but only changes in positive reappraisal and in self-distracting had an influence on depressive symptoms.

